# Semaglutide Adherence and Cardiovascular Diseases in Patients With Type 2 Diabetes: Evidence From Real-World Data in Japan

**DOI:** 10.7759/cureus.80511

**Published:** 2025-03-13

**Authors:** Chikako Masudo, Takeshi Horii, Ririka Suzuki, Kiyoshi Mihara

**Affiliations:** 1 Faculty of Pharmacy, Musashino University, Tokyo, JPN

**Keywords:** adherence, cardiovascular diseases, real-world, semaglutide, type 2 diabetes

## Abstract

Semaglutide, a glucagon-like peptide-1 receptor agonist (GLP-1RA) known for its glucose-lowering and weight-reducing effects, has been linked to reduced cardiovascular disease (CVD) risk in patients with type 2 diabetes (T2D). However, the impact of semaglutide adherence on CVD events in real-world settings remains unclear. This retrospective observational study analyzed Japanese claims data to investigate the relationship between semaglutide adherence, measured as the proportion of days covered (PDC), and CVD risk in patients with T2D. Good adherence was defined as PDC ≥0.8. The hazard ratio (HR) for CVD incidence in patients with PDC <0.8 was significantly higher than that of those with PDC ≥0.8 (HR: 1.77, 95% confidence interval (CI): 1.25-2.49). Female patients and those taking biguanides exhibited significantly lower HR for CVD (HR: 0.37 and HR: 0.53, respectively). No significant difference in HR was observed based on semaglutide formulation. The incidence of CVD in patients on semaglutide monotherapy with PDC <0.8 tended to be higher than in those with PDC ≥0.8 (HR: 1.56, 95% CI: 0.53-4.57), although no significant difference was found. These findings suggest that maintaining good adherence to semaglutide is important for reducing CVD risk and potentially improving clinical outcomes in patients with T2D, regardless of formulation.

## Introduction

Semaglutide, a glucagon-like peptide-1 receptor agonist (GLP-1RA), has been strongly recommended by the 2024 American Diabetes Association Standards of Care in Diabetes for type 2 diabetes (T2D) treatment due to its efficacy in lowering blood glucose levels and promoting significant weight reduction [1]. GLP-1RAs are recommended as first-line therapy for patients with established atherosclerotic cardiovascular disease (CVD) or those at high risk for CVD and chronic kidney disease, independent of baseline hemoglobin A1C (HbA1c), individualized HbA1c target, or metformin use. Large-scale randomized controlled trials have demonstrated the benefits of GLP-1RAs in reducing CVD risk and potentially preventing cerebral infarctions [2-5]. Cardiovascular disease is the primary cause of death in patients with T2D. Therefore, reducing CVD risk is crucial for T2D treatment [6]. Additionally, semaglutide is known for its potent weight-loss effects and is a Food and Drug Administration-approved treatment for obesity. However, side effects such as nausea, vomiting, and a decrease in appetite have been observed. Semaglutide is classified as a new GLP-1RA and has a long-acting formulation, requiring less frequent dosing. In this class, semaglutide is the only drug currently available in both injectable and oral formulations for T2D management. Adherence is identified as one of the factors that can determine the effectiveness of injectable GLP-1RAs [7]. While oral semaglutide (O-SEMA) offers convenience, its complex dosing and administration requirements may complicate daily use [8]. Oral semaglutide is required to be taken on an empty stomach, with a 30-minute post-dose interval before eating or taking other medications to ensure adequate drug absorption. Conversely, some patients prefer oral medications over injectables [8,9], which has been associated with decreased adherence to subcutaneous semaglutide (SEMA-SC) use. Since published clinical trials typically reflect outcomes in adherent patients, the impact of poor adherence on CVD incidence in real-world settings remains unclear. The relationship between medication adherence and CVD risk with two different formulations of the same active ingredient of GLP-1RA in patients with T2D has not been elucidated. Therefore, this study aimed to investigate the association between semaglutide adherence and CVD risk using real-world data from Japan and to clarify whether lower adherence is associated with higher CVD risk.

## Materials and methods

Study design

This retrospective cohort study utilized administrative claims data from April 1, 2008, to December 31, 2022. The data were obtained from the Medical Data Vision (MDV) database, which comprises medical insurance claim data from Diagnosis Procedure Combination (DPC) hospitals managed by Medical Data Vision Co., Ltd. (Tokyo, Japan) [10]. The database contains approximately 39.4 million inpatient and outpatient records from 463 Japanese DPC hospitals, with approximately 35% of patients aged ≥65 years [10]. The observation period commenced on the date of the initial semaglutide prescription.

Study population

**Figure 1:**
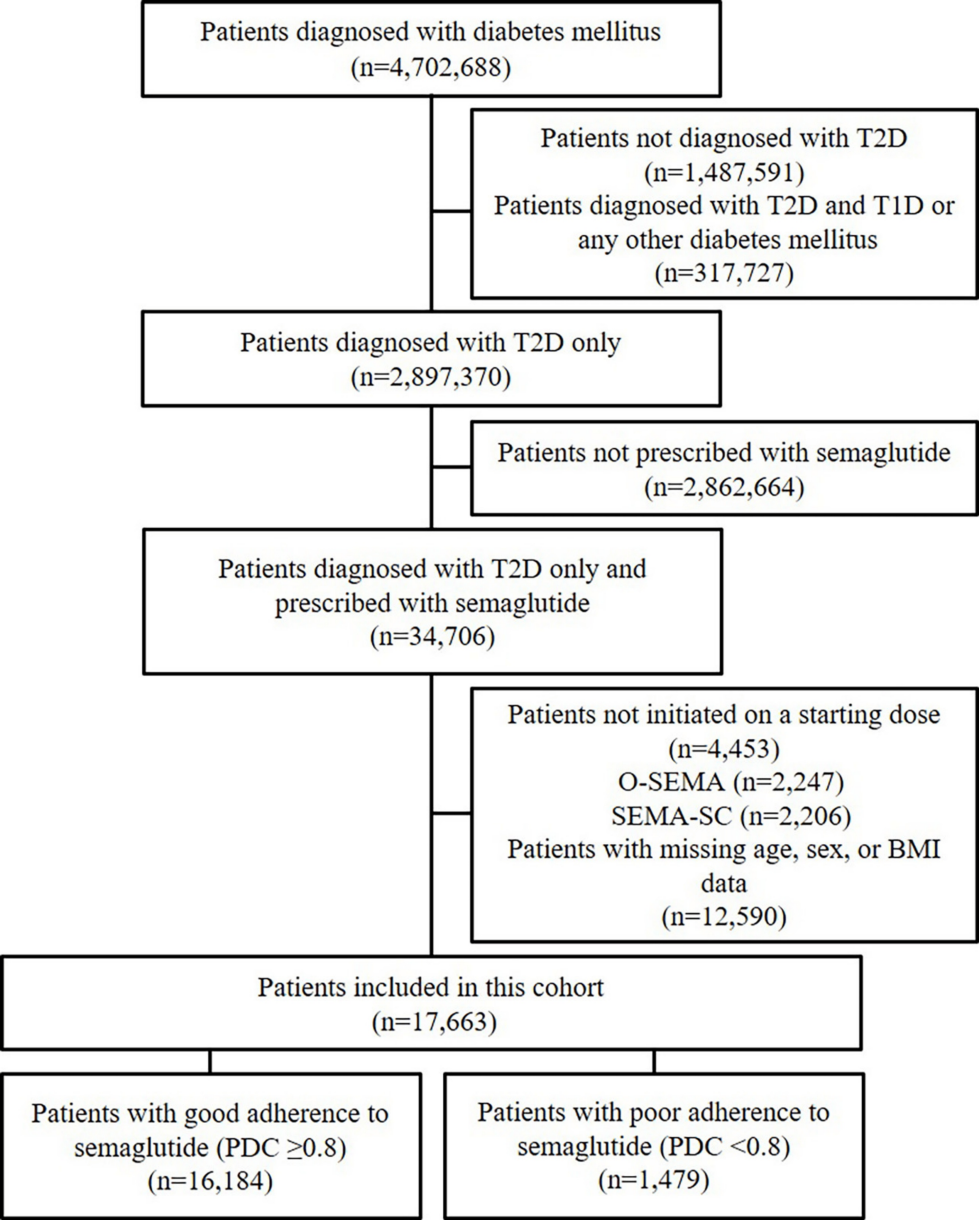
Flowchart showing patient selection T2D: type 2 diabetes; T1D: type 1 diabetes; O-SEMA: oral semaglutide; SEMA-SC: once-weekly semaglutide subcutaneous injection; PDC: proportion of days covered

The MDV database, containing data on 4,702,688 registered patients, was used to identify those diagnosed with T2D (International Classification of Diseases, Tenth Revision (ICD-10) codes: E10-E14) [11]. Figure 1 illustrates the participant selection process. The inclusion criteria for this study were (1) patients diagnosed with T2D and (2) patients prescribed semaglutide. The exclusion criteria were (1) patients not diagnosed with T2D; (2) patients not initiated on the starting dose of semaglutide (3 mg/day for O-SEMA and 0.25 mg/week for SEMA-SC); and (3) patients with missing age, sex, or body mass index (BMI) data. No age restrictions were applied; however, since semaglutide is approved for individuals aged ≥15 years in Japan, only patients aged ≥15 years were included in this study. Included patients were classified as having good adherence (proportion of days covered (PDC) ≥0.8, n=16,184) and poor adherence (PDC <0.8, n=1,479).

Identiﬁcation of outcomes

The primary outcomes were composite endpoints, including angina pectoris (AP) (ICD-10 codes: I20.0, I24.9), non-fatal myocardial infarction (NMI) (ICD-10 codes: I21x, I22x, I23x, I24.1), and non-fatal stroke (NS) (ICD-10 codes: I63x) in 2008-2012. Composite endpoints were defined as the occurrence of any AP or NMI events. Each outcome was identified using the ICD-10 codes [11]. As this study focused on arteriosclerosis, heart failure was excluded from the outcomes. Moreover, factors associated with non-adherence to semaglutide during the one-year observation period were investigated.

Medication adherence was assessed using PDC over a one-year period, which was calculated as PDC = (number of days with semaglutide access during the observation period / total number of days in the observation period); PDC ≥0.8 indicated good adherence, whereas PDC <0.8 indicated poor adherence [12-14]. For SEMA-SC, the calculation was based on a single dose lasting for seven days.

Patient characteristics

Baseline characteristics, including age, sex, and BMI, were obtained from billing records. Obesity was categorized based on Japanese guidelines (BMI <25 vs. BMI ≥25 kg/m²) [15].

Statistical analysis

Continuous variables were analyzed using the Student's t-test or Mann-Whitney U test, and results were reported as mean ± standard deviation. Categorical variables were analyzed using the Chi-square test or Fisher’s exact test and reported as absolute numbers or percentages. Event-free survival was estimated using the Kaplan-Meier method, and differences between groups were evaluated using the log-rank test. Hazard ratios (HRs) and 95% confidence intervals (CIs) for outcomes were analyzed using Cox proportional hazards regression models, with multivariate models incorporating variables such as sex, age, BMI, and medications. All statistical analyses were performed using IBM SPSS Statistics for Windows (version 29.0.0.0 (240); IBM Corp., Armonk, NY, USA), and statistical significance was set at p < 0.05.

## Results

Table 1 presents the baseline characteristics of patients with good and poor adherence. While significant differences in age, BMI, insulin combination, semaglutide monotherapy, and triple therapy were observed, there were no significant differences between the groups for other parameters. Dual therapy was primarily combined with SGLT2 inhibitors (1,466 patients), biguanides (1,233 patients), or insulin (1,223 patients), while triple therapy was mainly combined with biguanides (4,071 patients), sodium-glucose cotransporter 2 inhibitors (SGLT2is; 3,967 patients), or insulin (1,944 patients). Notably, O-SEMA demonstrated superior adherence and continuation rates compared to SEMA-SC.

**Table 1:** Baseline clinical characteristics and incidence of cardiovascular diseases in patients with type 2 diabetes prescribed with semaglutide Data are presented as numbers and percentages or means (standard deviation). The p-value was calculated for the difference between patients with PDC ≥0.8 and those with PDC <0.8. Obesity was classified according to the Japanese guidelines as BMI ≥25 kg/m². Continuous variables were analyzed using the Student's t-test*, while categorical variables were analyzed using the Chi-square test**. PDC: proportion of days covered; BMI: body mass index; O-SEMA: oral semaglutide; SEMA-SC: once-weekly semaglutide subcutaneous injection; α-GI: alpha-glucosidase inhibitor; SGLT2is: sodium-glucose cotransporter 2 inhibitors; CVD: cardiovascular diseases

Parameters	Overall (n=17,663)		PDC ≥0.8 (n=16,184)		PDC <0.8 (n=1,479)		Statistic	P-value
	n	%	n	%	n	%		
Male	10,493	59.41	9,621	59.44	872	58.96	0.13^**^	0.714
Female	7,170	40.59	6,563	40.55	607	41.04		
Age (years)	61.26±13.80		61.37±13.77		60.05±14.05		3.48^*^	<0.001
<65	9,679	54.80	8,814	54.46	865	58.49	11.56^**^	0.003
65-75	4,984	28.22	4,581	28.30	403	27.25		
≥75	3,000	16.98	2,789	17.23	211	14.27		
BMI (kg/m^2^)	29.08±6.23		29.02±6.21		29.67±6.46		-3.71^*^	<0.001
<25	4,621	26.16	4,271	26.39	350	23.66	5.21^**^	0.022
≥25	13,042	73.84	11,913	73.61	1,129	76.34		
O-SEMA	10,977	62.15	10,194	62.98	783	52.94	58.15^**^	<0.001
SEMA-SC	6,686	37.85	5,990	37.01	696	47.06		
α-GI	2,475	14.01	2,279	14.08	196	13.25	0.77^**^	0.379
Insulin	6,047	34.24	5,492	33.93	555	37.53	7.76^**^	0.005
Biguanide	1,0302	58.33	9,459	58.44	843	57.00	1.17^**^	0.279
SGLT2is	1,0293	58.27	9,441	58.33	852	57.61	0.30^**^	0.586
Sulfonylureas	3,066	17.36	2,830	17.49	236	15.96	2.21^**^	0.137
Glinides	2,114	11.97	1,942	12.00	172	11.63	0.18^**^	0.675
Thiazolidinediones	1,239	7.01	1,138	7.03	101	6.83	0.08^**^	0.770
Imeglimine	65	0.37	61	0.38	4	0.27	0.42^**^	0.517
Semaglutide monotherapy	1,524	8.63	1,376	8.50	148	10.01	6.55^**^	0.010
Dual therapy	4,430	25.08	4,056	25.06	374	25.29	3.03^**^	0.082
Triple therapy	5,972	33.81	5,504	34.01	468	31.64	5.24^**^	0.022
Four or more medications	5,737	32.48	5,248	32.43	489	33.06	3.17^**^	0.075
Angina pectoris	161	0.91	132	0.82	29	1.96	19.68^**^	<0.001
Myocardial infarction	43	0.24	36	0.22	7	0.47	3.51^**^	0.060
Strokes	43	0.24	39	0.24	4	0.27	0.05^**^	0.826
Total CVDs	245	1.39	206	1.27	39	2.64	18.43^**^	<0.001

**Figure 2:**
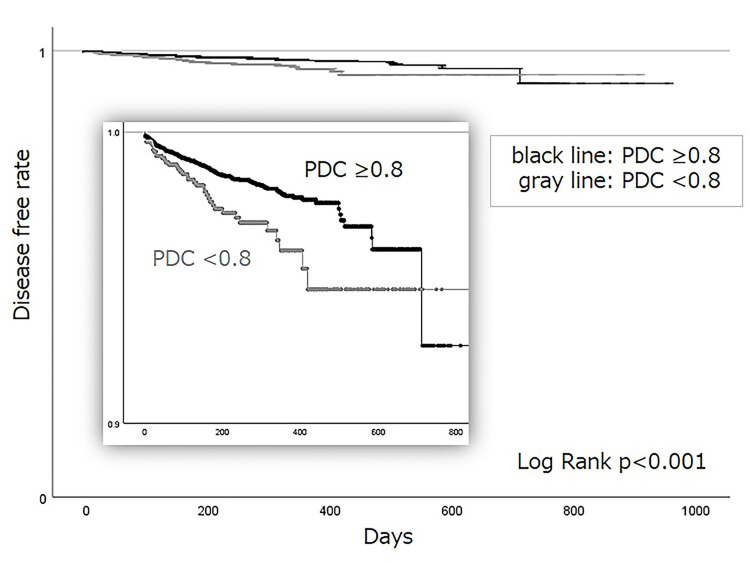
Kaplan–Meier analysis of the incidence of cardiovascular diseases in patients with type 2 diabetes prescribed semaglutide during the one-year observation period. PDC: proportion of days covered

Kaplan-Meier analysis evaluated CVD incidence during the observation period between patients with good and poor adherence (Figure 2).

**Figure 3:**
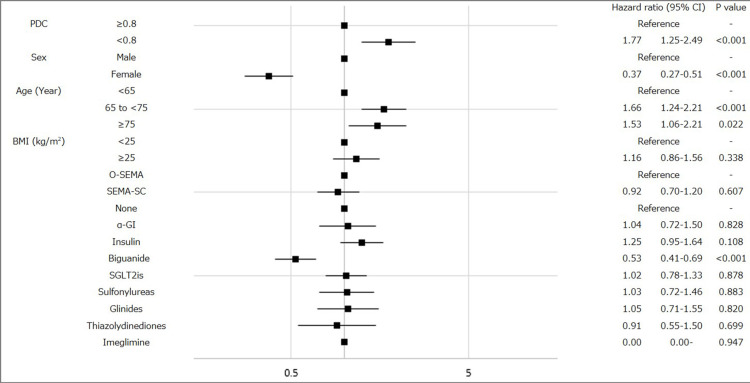
Forest plots showing the hazard ratio (HR) for cardiovascular diseases (CVD) in patients with type 2 diabetes (T2D) who were prescribed semaglutide according to baseline clinical characteristics: HR for CVD in all patients with T2D prescribed semaglutide. Squares represent HRs, and horizontal bars indicate the 95% confidence intervals (CIs) of estimated HRs. PDC: proportion of days covered; BMI: body mass index; O-SEMA: oral semaglutide; SEMA-SC: once-weekly semaglutide subcutaneous injection; α-GI: alpha-glucosidase inhibitor; SGLT2is: sodium–glucose cotransporter 2 inhibitors

Patients with PDC <0.8 showed a significantly higher cumulative incidence of CVD events compared to those with PDC ≥0.8 (p=0.001). The HR for cumulative CVD with PDC <0.8 was 1.77 (95% CI: 1.25-2.49), which was significantly different from that of the reference group (PDC ≥0.8) (Figure 3). The incidence of CVD was also evaluated according to baseline clinical characteristics. Female patients and those taking biguanides exhibited significantly lower HRs for CVD (HR: 0.37 and HR: 0.53, respectively; Figure 3). In contrast, patients aged 65-75 years and ≥75 years demonstrated significantly higher HRs compared to those aged <65 (HR: 1.66 and HR: 1.53, respectively; Figure 3). Additionally, no significant differences in CVD incidence were observed based on semaglutide formulation, alpha-glucosidase inhibitor (α-GI), insulin, SGLT2is, sulfonylureas, glinides, thiazolidinediones, and imeglimin.

**Figure 4:**
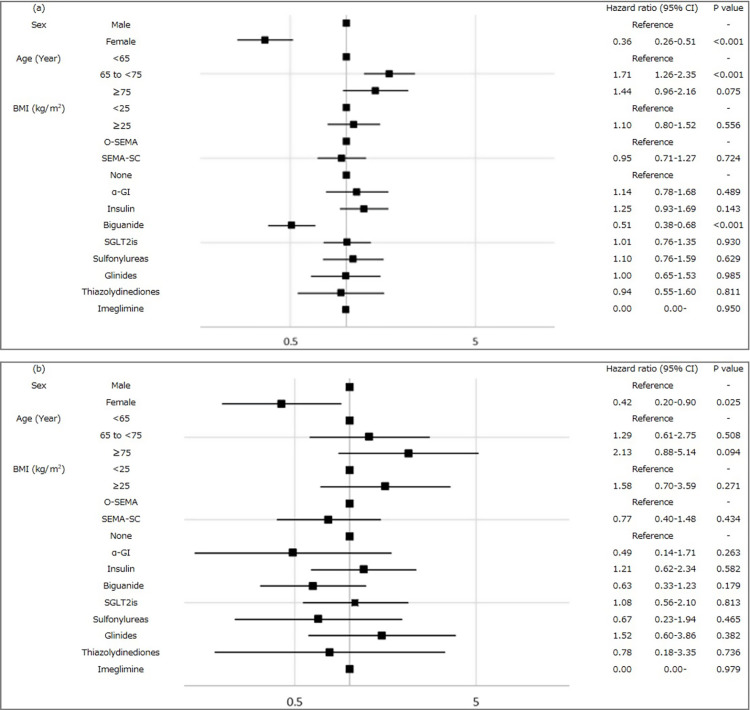
Forest plots showing the hazard ratio (HR) for cardiovascular diseases (CVD) in patients with type 2 diabetes (T2D) who were prescribed semaglutide according to baseline clinical characteristics: (a) HR for CVD in patients with PDC ≥0.8. (b) HR for CVD with patients with PDC <0.8. Squares represent HRs, and horizontal bars indicate the 95% confidence intervals (CIs) of estimated HRs. PDC: proportion of days covered; BMI: body mass index; O-SEMA: oral semaglutide; SEMA-SC: once-weekly semaglutide subcutaneous injection; α-GI: alpha-glucosidase inhibitor; SGLT2is: sodium–glucose cotransporter 2 inhibitors

Given that 16,184 (92%) patients had a PDC ≥0.8, the results predominantly reflect outcomes in patients with good adherence. Nevertheless, similar overall trends were observed in both groups (Figures 4a-4b).

We also examined the incidence of CVD in patients on semaglutide monotherapy. The HR for cumulative CVD with PDC <0.8 was 1.56 (95% CI: 0.53-4.57). Although the difference was not statistically significant, the HR tended to be higher than that with PDC ≥0.8. The CVD event incidence rate with PDC <0.8 was 2.70%, approximately 1.69 times higher than that with PDC ≥0.8 (1.60%). This result demonstrated a similar trend to that observed for patients receiving combination therapy.

## Discussion

In this study, good adherence to semaglutide (PDC ≥0.8) was associated with lower CVD risk and potentially improved clinical outcomes in patients with T2D. Although several previous studies have demonstrated the association between adherence and CVD risk in T2D patients [13,16,17], they did not assess this with two different formulations of the same active ingredient of GLP-1RA in patients with T2D, which has not been elucidated.

This finding may be attributed to insufficient understanding of semaglutide's benefits or inadequate management of other lifestyle factors when adherence is low [16,17]. Furthermore, O-SEMA was associated with better adherence compared to SEMA-SC, likely due to its non-invasive administration [9,18,19]. Interestingly, no significant difference in event rates was observed between the two formulations. This may be explained by the high average PDC (>90%) that was achieved with both formulations over the one-year observation period, suggesting that good adherence is attainable with either route [20,21]. Given these findings, selecting the appropriate formulation should be tailored to individual patient preferences and needs.

Regarding baseline characteristics, male sex was significantly associated with CVD risk. Previous research has shown that anginas are more prevalent in male patients than in female patients [22,23]. Since 161 (66%) of the CVD events in this study were angina pectoris, this finding may reflect this trend. Conversely, although the patient population was slightly younger than those taking other antidiabetic medications (e.g., mean patient age of 59.4 years for biguanides, 60.9 years for insulin, and 60.2 years for SGLT2is), the use of biguanides was associated with a lower incidence of CVD, which is consistent with previous studies [24,25]. In addition, patients aged >65 years had a higher risk of CVD than those aged <65 years, likely reflecting the association between aging and increased risk [26,27]. The absence of significant differences in the HRs of patients with semaglutide monotherapy (n=1,524) may be attributed to the relatively small sample size.

Despite the insights provided in this study, one significant limitation was the lack of data on potential confounding factors, such as comorbidities and concomitant medications. This study was a retrospective observational study conducted using Japanese claims data, and the findings may not be generalizable to other populations.

## Conclusions

Good adherence to semaglutide was associated with a lower incidence of CVD compared to poor adherence in Japanese patients with T2D. The risk of CVD was reduced in female patients and those taking biguanides, while it increased in patients aged 65 years and older. Additionally, no significant difference in CVD incidence was observed between the formulations of semaglutide. The findings of this cohort study provide valuable insights that can aid in optimizing semaglutide-based treatment strategies to improve clinical outcomes in this patient population.
